# Stronger than Ever: Multifilament Fiberglass Posts Boost Maxillary Premolar Fracture Resistance

**DOI:** 10.3390/jcm12082975

**Published:** 2023-04-19

**Authors:** Naji Kharouf, Eugenio Pedullà, Gianluca Plotino, Hamdi Jmal, Mohammed-El-Habib Alloui, Philippine Simonis, Patrice Laquerriere, Valentina Macaluso, Dina Abdellatif, Raphaël Richert, Youssef Haikel, Davide Mancino

**Affiliations:** 1 Department of Biomaterials and Bioengineering, INSERM UMR_S, Strasbourg University, 67000 Strasbourg, Franceyoussef.haikel@unistra.fr (Y.H.);; 2 Department of Endodontics, Faculty of Dental Medicine, Strasbourg University, 67000 Strasbourg, France; philippine.simonis@gmail.com; 3 Department of General Surgery and Medical Surgical Specialties, University of Catania, 95128 Catania, Italy; eugeniopedulla@gmail.com; 4 Private Practice, Grande Plotino & Torsello—Studio di Odontoiatria, 00187 Rome, Italy; endo@gianlucaplotino.com; 5 ICube Laboratory, UMR 7357 CNRS, Mechanics Department, University of Strasbourg, 67000 Strasbourg, France; 6 Institute Pluridisciplinaire Hubert CURIEN (IPHC), 67000 Strasbourg, France; patrice.laquerriere@iphc.cnrs.fr; 7 ESTA, School of Business and Technology, 90000 Belfort, France; vmacaluso@esta-groupe.fr; 8 Department of Endodontics, Alexandria University, Alexandria 5424041, Egypt; dinaabdellatif@gmail.com; 9 Hospices Civils de Lyon, PAM Odontologie, 69100 Lyon, France; richertg@gmail.com; 10 Laboratoire de Mécanique des Contacts et Structures, UMR 5259 CNRS/INSA Lyon, 69100 Lyon, France; 11 Pôle de Médecine et Chirurgie Bucco-Dentaire, Hôpital Civil, Hôpitaux Universitaire de Strasbourg, 67000 Strasbourg, France

**Keywords:** multifilament fiberglass posts, fracture resistance, single fiber post

## Abstract

This paper investigates the influence of cavity configuration and post-endodontic restoration on the fracture resistance, failure mode and stress distribution of premolars by using a method of fracture failure test and finite elements analysis (FEA) coupled to Weibull analysis (WA). One hundred premolars were divided into one control group (G_contr_) (*n* = 10) and three experimental groups, according to the post-endodontic restoration (*n* = 30), G_1_, restored using composite, G_2_, restored using single fiber post and G_3_, restored using multifilament fiberglass posts (m-FGP) without post-space preparation. Each experimental group was divided into three subgroups according to the type of coronal cavity configuration (*n* = 10): G_1O_, G_2O,_ and G_3O_ with occlusal (O) cavity configuration; G_1MO_, G_2MO_, and G_3MO_ with mesio-occlusal (MO); and G_1MOD_, G_2MOD_, and G_3MOD_ with mesio-occluso-distal (MOD). After thermomechanical aging, all the specimens were tested under compression load, and failure mode was determined. FEA and WA supplemented destructive tests. Data were statistically analyzed. Irrespective of residual tooth substance, G_1_ and G_2_ exhibited lower fracture resistance than G_contr_ (*p* < 0.05), whereas G_3_ showed no difference compared to G_contr_ (*p* > 0.05). Regarding the type of restoration, no difference was highlighted between G_1O_ and G_2O,_ G_1MO_ and G_2MO,_ or G_1MOD_ and G_2MOD_ (*p* > 0.05), whereas G_3O,_ G_3MO,_ and G_3MOD_ exhibit higher fracture resistance (*p* < 0.05) than G_1O_ and G_2O_, G_1MO_ and G_2MO_, and G_1MOD_ and G_2MOD_, respectively. Regarding cavity configuration: in G_1_ and G_2,_ G_1O_ and G_2O_ exhibited higher fracture resistance than G_1MOD_ and G_2MOD_, respectively (*p* < 0.05). In G_3_, there was no difference among G_3O_, G_3MO_ and G_3MOD_ (*p* > 0.05). No difference was found among the different groups and subgroups regarding the failure mode. After aging, premolars restored with multifilament fiberglass posts demonstrated fracture resistance values comparable to those of an intact tooth, irrespective of the different type of cavity configuration.

## 1. Introduction

Endodontically treated teeth (ETT) are widely perceived as more brittle and more prone to fracture than vital teeth. Their vulnerability is mainly due to the volumetric loss of hard tissues [1,2,3] and probably to the endodontic treatment itself [4,5]. Since the structure and composition of teeth are perfectly adapted to the functional needs of the mouth and are superior to any artificial material [6], the guiding principle for endodontic and post-endodontic restorations should be to remove the least possible amount of sound tissue. As a consequence, root canal treatment and post-endodontic restoration should be performed under magnification to avoid unnecessary healthy tissue sacrifice.

Restoration materials should mimic as closely as possible the characteristics of the lost tissues [1,6] to achieve a uniform distribution of stress on the residual tooth structure under mastication [1,2,3,4,5,7,8,9,10]. However, post-endodontic restoration may represent a real challenge both for structurally compromised teeth and for those with more residual tooth structure; several studies have established that restorative complications represent the main reason for failure of ETT, which may lead to tooth extraction [11,12].

For many years, intracanal posts were proposed to restore ETT in order to reduce the failure rate of post-endodontic restorations. Their use would increase the retention of coronal restorations. Although some past studies have shown that the post would increase the fracture resistance of EET [13,14,15], other studies claim the opposite [16].

Although there are plenty of different intracanal post materials, in order to reduce the occurrence of unrestorable root fractures, the use of fiber posts with mechanical characteristics similar to those of dentine have been suggested since more than 30 years [13,17]. In fact, it has been reported that the use of fiber posts would be successful in decreasing the incidence of fractures in ETT [5,18]. However, it has been strongly recommended that the post should be placed inside the root canal without sacrificing any further sound root dentin [19] to avoid adversely affecting the residual tooth structure [20,21]. In fact, post-space preparation may excessively reduce the thickness of the root walls, especially in oval canals [22], thus increasing the risk for root fracture [23]. In this regard, the results of a past study show that the length of the fiber post does not influence the fracture resistance of ETT [24] and post-space should be prepared to be about 1/3 of the working length.

Biomechanically, post-space preparation should just be limited to clean the canal walls from the smear layer, smear plug and possible residual filling materials without any additional dentin sacrifice after the endodontic treatment. Therefore, posts that adapt to the canal diameter at the end of the endodontic treatment should be used in order to follow a minimally invasive intracanal protocol [25]. Although the literature is not unanimous on the benefit of using posts to restore ETT [26], a randomized clinical trial [27] concluded that the placement of a fiber post was a significant factor for tooth survival and restorative success in endodontically treated premolars with different levels of coronal tissue loss. Moreover, several systematic reviews reported that the use of a fiber post with direct composite restorations could ameliorate the fracture resistance of maxillary premolars ETT [15]. Similarly, a retrospective study [28] concluded that teeth restored with fiber posts had statistically higher success rates than teeth restored without posts, whether they were restored with a crown or not, over a mean follow-up period of 8.8 years. Moreover, according to a recent in vitro study, maxillary premolars restored with a fiber post showed a significant higher fracture resistance than direct restorations without any intra-radicular retention, regardless of the number of residual walls [25]. Several recent studies have also pointed out that in oval canals, the use of multiple small fiber posts, instead of a single post, led to better reinforcement and stress distribution [25,29], limiting the loss of root dentin during post-space preparation [17,30].

**Figure 1:**
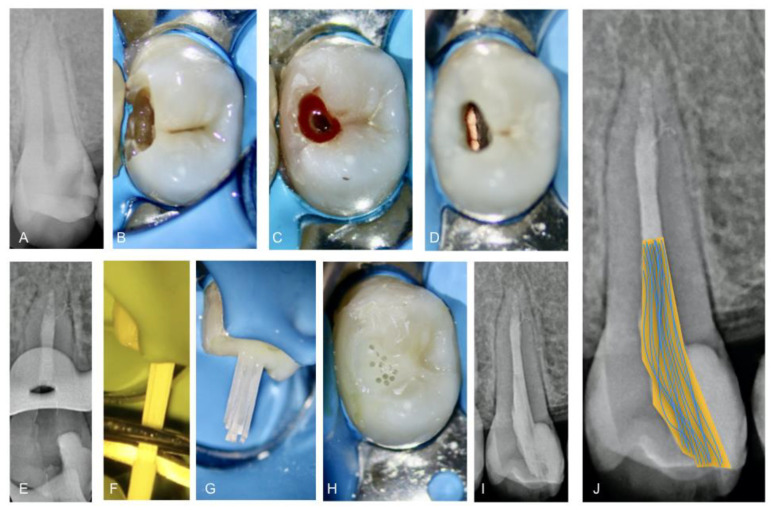
Root canal treatment and post-endodontic restoration of a second maxillary premolar diagnosed with symptomatic irreversible pulpitis: (**A**) pre-operative radiograph; (**B**) tooth under rubber dam isolation; (**C**) pre-endodontic restoration and driven access cavity preparation; (**D**) visualization of the flat-oval root canal after root canal filling; (**E**) intra-operative radiograph; (**F**) cutting the part of the multifilament fiberglass posts enveloped in the rubber ring; (**G**) clinical visualization after light curing; (**H**) clinical visualization after the end of restorative procedure; (**I**) post-operative radiograph; (**J**) trajectory schematization of multifilament fiberglass posts emphasizing their flexibility.

Recently, an intracanal retainer formed by independent multifilament fiberglass posts (m-FGP), also called micro fasciculated posts, have been launched on the market. They can be used without prior preparation of the post-space; therefore, the removal of root canal dentin may be minimized. According to the manufacturer (Bio Medical Components, Tullin, France), they are flexible, may adapt to any root canal anatomy and can be used when the access cavity is not on axis with the root canal orifice (Figure 1).

Therefore, the main aim of the present ex vivo study was to investigate the influence of cavity configuration and post-endodontic restoration on the fracture resistance, failure mode and stress distribution of premolars by using a method of fracture failure test and finite elements analysis (FEA) coupled to Weibull analysis (WA). Limited studies investigate the risk of fracture of these strategies in vitro and in silico. In comparison, Weibull analysis has commonly been used in determining the likelihood of fracture in premolars reinforced with fibers [31]. This method has proven effective in predicting the probability of cumulative failure at specific stress levels and has shown strong agreement with experimental findings [32]. The null hypotheses tested were that the fracture resistance and the failure mode did not differ according to the type of post-endodontic restoration, to the cavity configuration and to the type of post-endodontic restoration.

## 2. Materials and Methods

### 2.1. Specimen Preparations

Two hundred maxillary first permanent premolars extracted for orthodontic reasons with fully formed roots and a total length between 21 and 23 mm were collected under patient-informed consent. The protocol was approved by the Ethics Committee of the Medical, Odontology School, and Strasbourg University Hospital (Protocol No. CE-2019-05). Cone-beam computed tomography (CBCT) was used in the selection of the teeth respecting the following criteria: single canal, a long/short canal diameter ratio at 5 mm from the apex >2 [33], the length of root canal (orifice to apical foramen) set at 14 ± 1 mm, primary root curvature ≤ 20° in bucco-lingual and mesio-distal view [34], main curvature radius ≥ 4 mm.

After selection, 100 teeth were finally included in the experimental design. Premolars were randomly assigned to 1 control group (*n* = 10) consisting of intact teeth (G_contr_) and 3 experimental groups (*n* = 30) according to the type of post-endodontic restoration: composite without a post (G_1_), single fiber post after controlled post-space preparation (G_2_) and multifilament fiberglass posts (m-FGP) without post-space preparation (G_3_). These experimental groups were further divided into 3 subgroups (*n* = 10) according to the type of cavity configuration: occlusal (O), mesio-occlusal (MO) and mesio-occluso-distal (MOD).

All endodontic and restorative procedures were performed by the same experienced operator. After debridement of the root surface, specimens were stored in a 0.1% thymol solution at 4 °C. The access cavities were prepared using a high-speed headpiece (Kavo Dental GmbH, Biberach, Germany) using cylindrical diamond burs (#806314014; Komet, Schaumburg, IL, USA) under water-cooling, aiming to replicate the morphology of the pulp chamber roof. Then, 30 premolars underwent no additional preparation and remained with an occlusal cavity (O) configuration, 30 underwent a standardized mesio-occlusal (MO) cavity preparation and 30 underwent a standardized mesio-occluso-distal (MOD) cavity preparation. The residual thickness of buccal and lingual cusps at the height of the contour was 2.5 ± 0.2 mm, with the mesial and distal cervical margin located 1.5 mm coronal to the cement–enamel junction (CEJ).

A size 10 K-file was used to establish the working length under an operative microscope (Zumax Medical Co., Ltd., Suzhou, Jiangsu, China) by subtracting 1 mm from the length at which the tip of the instrument was visible at the apical foramen. The root canals were then instrumented with rotary nickel-titanium instruments (Plex V; Orodeka, Jining, Shandong, China), up to a tip size 30/.04 taper and irrigated with 3 mL of 5.25% sodium hypochlorite using a 31-gauge Navitip needle (Ultradent Products, South Jordan, UT, USA). Canals were then rinsed with distilled water, dried with absorbent paper points and filled by the combination of gutta-percha (Roeko, Langenau, Germany) and sealer (Sealapex, Kerr Endodontics, Gilbert, AZ, USA) using the continuous wave of condensation technique without performing the back filling (Fast-Pack Pro, Eighteeth, Changzhou City, Jiangsu Province, China), with the exception of the G_1_ groups without post-space preparation.

### 2.2. Restorative Procedures: Post Placement and Composite Restoration

The 90 prepared samples were divided into three experimental groups (*n* = 30) according to the type of post-endodontic restoration: Group 1 (G_1_), restored using composite without post (Ceram-X; Dentsply DeTrey, Hilpoltstein, Germany); Group 2 (G_2_), restored using a single fiber-reinforced composite (s-FRC) post (Bioligth; Tullins, France); Group 3 (G_3_), restored using multifilament fiberglass posts (m-FGP; Bioligth).

**Figure 2:**
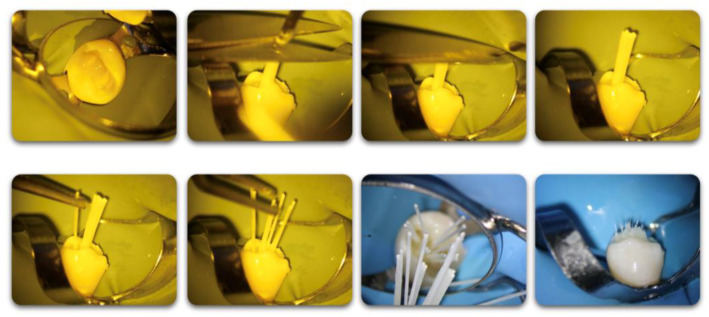
Multifilament fiberglass posts placement steps.

In group G_2_ (s-FRC), the post-space was prepared 5 ± 1 mm shorter than the working length, using a 1.2 drill (Bioligth) (1.5 N.cm, 2000 rpm), which was compatible with the diameter of the glass fiber post used (apical diameter 0.65 mm, coronal diameter 1.2 mm). Drills were replaced every 5 samples. The canal was etched using liquid 37% phosphoric acid for 60 s [35], rinsed with distilled water and dried with a gentle blowing air and then with paper points. The post was then cemented using a dual-cure universal adhesive system and cement (Clearfil core build-up kit; Kuraray Europe GMBH, Troisdorf, Germany) in accordance with the manufacturer’s instructions. In group G_3_ (m-FGP), no post space preparation was required, and the part of the canal coronal to the 5 mm of apical gutta-percha was cleaned using Versa Brush (Vista Apex, Vista Dental Products, WI, USA) (1.5 N.cm, 500 rpm) under water cooling. The canal was etched, rinsed and dried as in group G_2_. After delivering the resin cement into the canal as in group G_2_, the m-FGP was inserted with tweezers into the root canal at a depth of 3 mm up to the root canal orifice. Then, the colored rubber sleeve, gripping the multifilament fiberglass posts as a unit, was cut using a scissor and micro-posts, now being independent, were deeply rooted one by one using a plugger. The coronal part of each filament was positioned in different directions (Figure 2).

In MO and MOD groups, the mesial and distal walls were previously restored using a universal adhesive (Prime&Bond XP; Dentsply DeTrey, Hilpoltstein, Germany) and resin composites (Ceram-X; Dentsply DeTrey, Hilpoltstein, Germany). G_1_ groups were restored in the same way, but no posts were used.

In order to simulate a 0.2–0.3 mm thick periodontal ligament (PDL), each root was immersed in melted wax-up to 2 mm apical to CEJ [36]. A silicone cubic mold (25 × 25 × 25 mm^3^) was used to embed all the specimens in acrylic self-curing resin (OrthocrylEQ; Dentaurum, Ispringen, Germany) up to 2 mm apical to the CEJ. Each root was removed from the resin block when primary signs of polymerization were noticed. The wax layer was removed with hot water and then replaced by a silicone-based impression material (Aquasil Ultra XLV; Dentsply DeTrey, Hilpoltstein, Germany), which was injected into the acrylic resin block prior to reinsertion of the specimen.

### 2.3. Thermomechanical Aging

The samples were submitted to thermocycling challenge using a thermo-cycling machine (Customized machine) programmed to perform 12,000 thermocycles during two weeks at temperatures between 5 and 55 °C, with a dwell time of 30 s at each bath temperature.

The mechanical fatigue was then completed in a dynamic testing machine (Instron; Electropuls 10,000, High Wycombe, UK). A metal device was fabricated by ICube laboratory (Strasbourg, France). It allowed fixing the specimen at an angle of 45° between a stainless-steel spherical antagonist (diameter of 6 mm) and the tooth axis, with contact on the center of the mesio-distal groove. A total of 20,000 cycles of sinusoidal force load with an amplitude of 22.5 Newtons (N) were applied to each specimen at a frequency of 2 Hz [5,37]. The cyclic ratio is equal to Fmin/Fmax = 5 N/50 N = 0.1 [5,37]. During the test, the force was recorded using a dynamic force cell (10 kN) and treated with Matrix software (Instron, High Wycombe, UK).

### 2.4. Determination of Load Resistance

After fatigue load, each specimen was submitted to a quasi-static load until fracture using the same testing machine (Instron; Electropuls 10,000, High Wycombe, UK). The same stainless steel spherical antagonist was used to load the samples under 45° oblique compression conditions until failure [38] at a crosshead speed of 1 mm/min. The force was applied on the center of the mesio-distal groove. Through the dynamic force cell sensor, a sudden decrease in force of more than 30 N was considered as an indication of failure. The maximum force up to this point was recorded as the force at fracture in N.

### 2.5. Evaluation of Fracture Patterns

All samples were then removed from their acrylic cube and assessed for failure mode evaluation using an optical numeric microscope (Keyence VHX5000, Osaka, Japan) at 100× magnification. Favorable failures were defined as repairable failures, including retention failures and fractures of the root above the level of bone simulation. Unfavorable failures were defined as irreparable failures as root fractures below the level of bone simulation.

**Figure 3:**
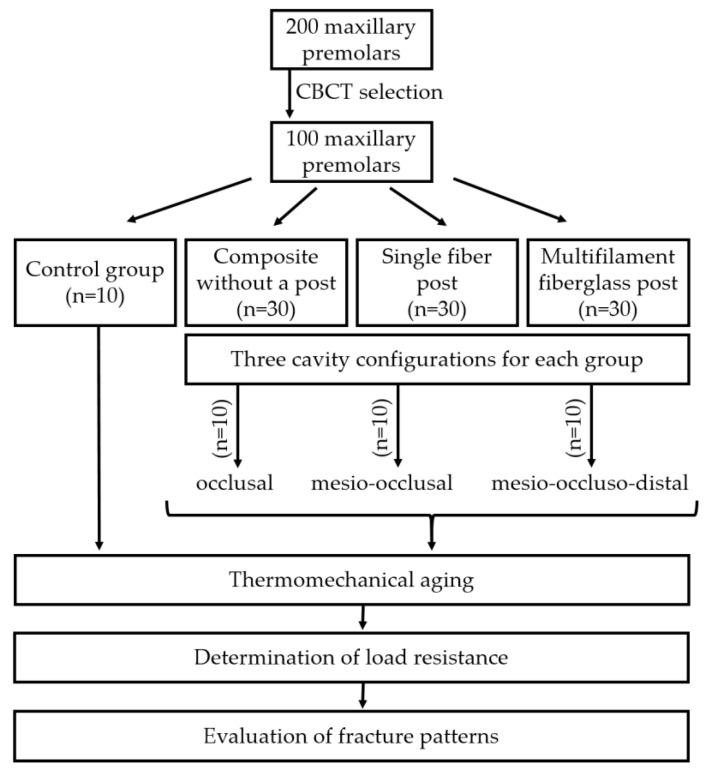
Flowchart of the methodological steps.

All the in vitro methodological steps are summarized in Figure 3.

### 2.6. Finite Element Analysis (FEA) and Weibull Analysis (WA)

An intact human maxillary premolar, extracted for orthodontic reasons, was scanned by using a µCT (IRIS, Inviscan, Strasbourg, France). The acquisition settings were 2000 projection (60 × 60 × 60 µm^3^ voxel size) at 80 kVp. The different anatomical structures were segmented based on a previously validated procedure [39]. The segmented 3D image was modified to model the three restorative strategies: no post, s-FRC and m-FGP for three clinical situations: occlusal, mesio-occlusal and mesio-occluso-distal cavities. The alveolar bone and a periodontal ligament of 0.2 mm were simulated around the root [40]. The segmented 3D image was then meshed using quadratic tetrahedral elements after a convergence test. All dental materials were supposed homogeneous, linearly elastic except from the periodontal ligament supposed hyper-elastic. The attributed material properties were referenced from the literature [40]. There was a perfect bonding between each component, and an oblique load of 300 N was applied on the vestibular cupid of the premolar to simulate masticatory forces. The nodes of the lateral faces of the mesial and distal cortical bone were constrained to prevent displacement following previous protocols [41,42]. Finite element analysis (FEA) was conducted on the software Abaqus (Dassault Systèmes, Vélizy-Villacoublay, France) to calculate the strain and von Mises stresses of the premolar.

The Weibull analysis (WA) was combined with FEA to assess the risk of fracture of dentin. Failure was assumed to occur from the maximum principal stress on stress concentration areas. The survival probability *P_s_* for a load F can be expressed as:

$$P_{s}\left(\sigma \right)=exp\left[-{\left(\frac{\sigma }{\sigma_{0}}\right)}^{m}\right]$$

where *σ* represents the maximum principal stress, *σ*_0_ represents the characteristic strength and m represents the Weibull modulus of the dentin.

### 2.7. Statistical Analysis

The Shapiro–Wilk test was used to verify the normality of data within all groups. One-Way Analysis of Variance on ranks (ANOVA) including Pairwise Multiple Comparison Procedures (Tukey test) was applied to determine whether significant differences existed in the fracture resistance values of the different groups. Data analyses were performed with Sigma Plot (11.2, Systat Software, Inc., San Jose, CA, USA). A Chi-square test was used to determine whether there were significant differences between the failure mode in the different groups. A significance level of *p* < 0.05 was adopted.

## 3. Results

The mean fracture load resistance and the standard deviation for the different experimental groups are summarized in Table 1. Irrespective to the cavity configuration, the G_1_ and G_2_ exhibited statistically lower fracture resistance than the G_contr_ (*p* < 0.05), whereas G_3_ showed no statistically significant difference compared to G_contr_ (*p* > 0.05).

No statistical difference was highlighted between G_1O_ and G_2O,_ G_1MO_ and G_2MO,_ G_1MOD_ and G_2MOD_, respectively (*p* > 0.05) with regard to the type of restoration, whereas G_3O,_ G_3MO,_ and G_3MOD_ exhibited statistically higher fracture resistance than G_1O_ and G_2O_, G_1MO_ and G_2MO_, and G_1MOD_ and G_2MOD_, respectively (*p* < 0.05).

Interaction analysis among subgroups with the same type of post-endodontic restoration revealed that G_1O_ and G_1MO_ exhibited statistically higher fracture resistance than G_1MOD_ (*p* < 0.05) with regard to the cavity configuration, whereas no difference was shown between G_1O_ and G_1MO_ (*p* > 0.05). G_2,_ G_2O_ exhibited statistically higher fracture resistance than G_2MOD_ (*p* < 0.05), whereas no difference was shown between G_2O_ and G_2MO_ and between G_2MO_ and G_2MOD_. In the G_3_ group, no statistical difference was shown among G_3O_, G_3MO_ and G_3MOD_ (*p* > 0.05).

**Figure 4:**
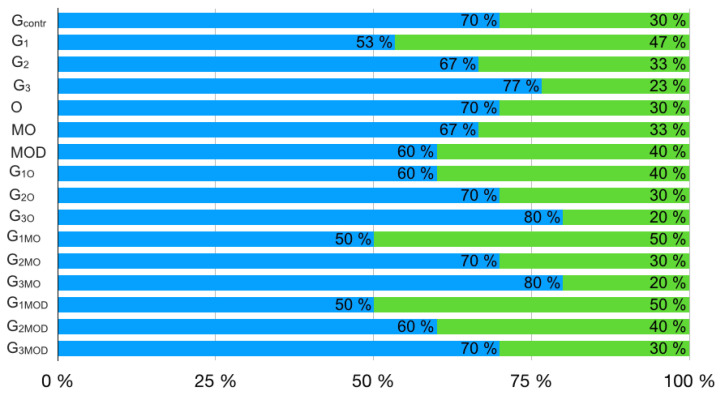
Percentage incidence of favorable (blue) and unfavorable (green) fractures.

Results for the failure mode of the different experimental groups and subgroups are summarized in Figure 4. No statistical difference among the different groups was observed.

**Figure 5:**
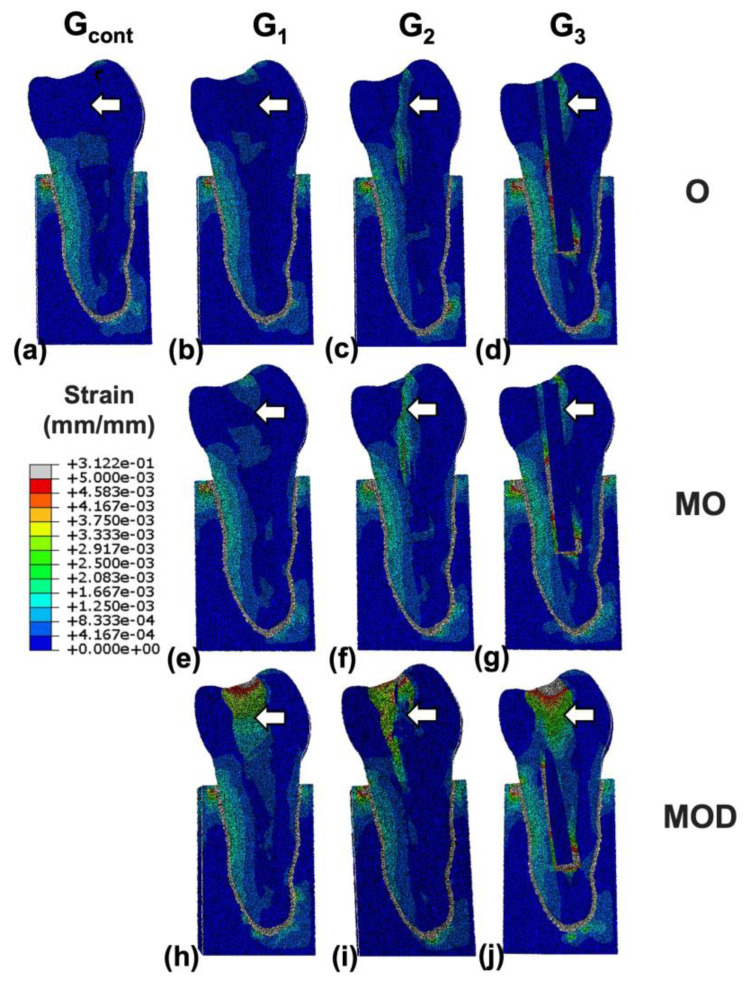
Strain distributions for finite element models representative of (**a**) intact, (**b**) occlusal (O) resin, (**c**) O multiple fiber-reinforced composite (mFRC), (**d**) O single fiber-reinforced composite (sFRC), (**e**) mesio-occlusal (MO) resin, (**f**) MO mFRC, (**g**) MO sFRC, (**h**) mesio-occluso-distal (MOD) resin reconstructions, (**i**) MOD mFRC and (**j**) MOD sFRC. Micro-fiber posts limit the transfer of stress in the resin in the radicular part in the contrary to sFRC, where strains are present all around the post (white arrows presenting different strains in the resin depending on the restorative choice).

Concerning FEA and WA analyses, samples with O and MO cavity configuration showed high strain in the lateral part of the root, while samples with MOD cavity configuration reported high strains on the coronal tooth structure, notably for G_1_ and G_2_ (Figure 5), with significantly higher cervical root stresses and resin strains than those with MO, O cavity configuration and intact teeth. G_2_ and G_3_ exhibited high strain around the post.

**Figure 6:**
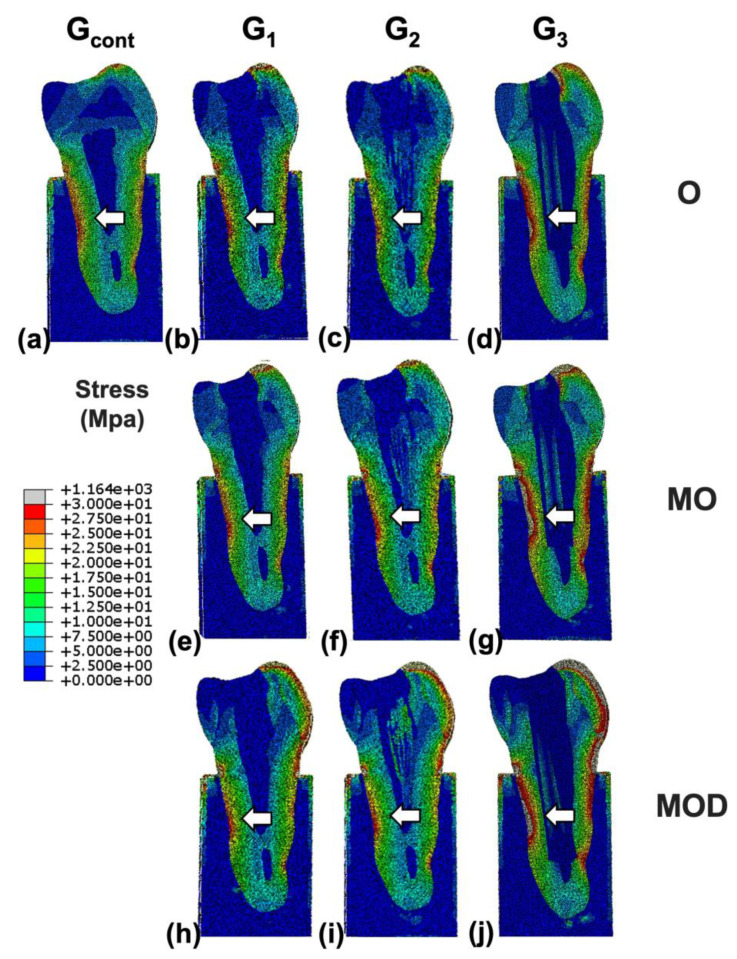
Stress distributions for finite element models representative of (**a**) intact, (**b**) occlusal (O) resin, (**c**) O multiple fiber-reinforced composite (mFRC), (**d**) O single fiber-reinforced composite (sFRC), (**e**) mesio-occlusal (MO) resin, (**f**) MO mFRC, (**g**) MO sFRC, (**h**) mesio-occluso-distal (MOD) resin reconstructions, (**i**) MOD mFRC and (**j**) MOD sFRC. A single post is present in the center of the root canal where stresses are low (white arrows are presenting different stress concentrations in the root depending on the restorative choice).

In addition, all samples including G_contr_ displayed high stresses on the lateral parts of the root with low stresses in the center of the root (Figure 6).

**Figure 7:**
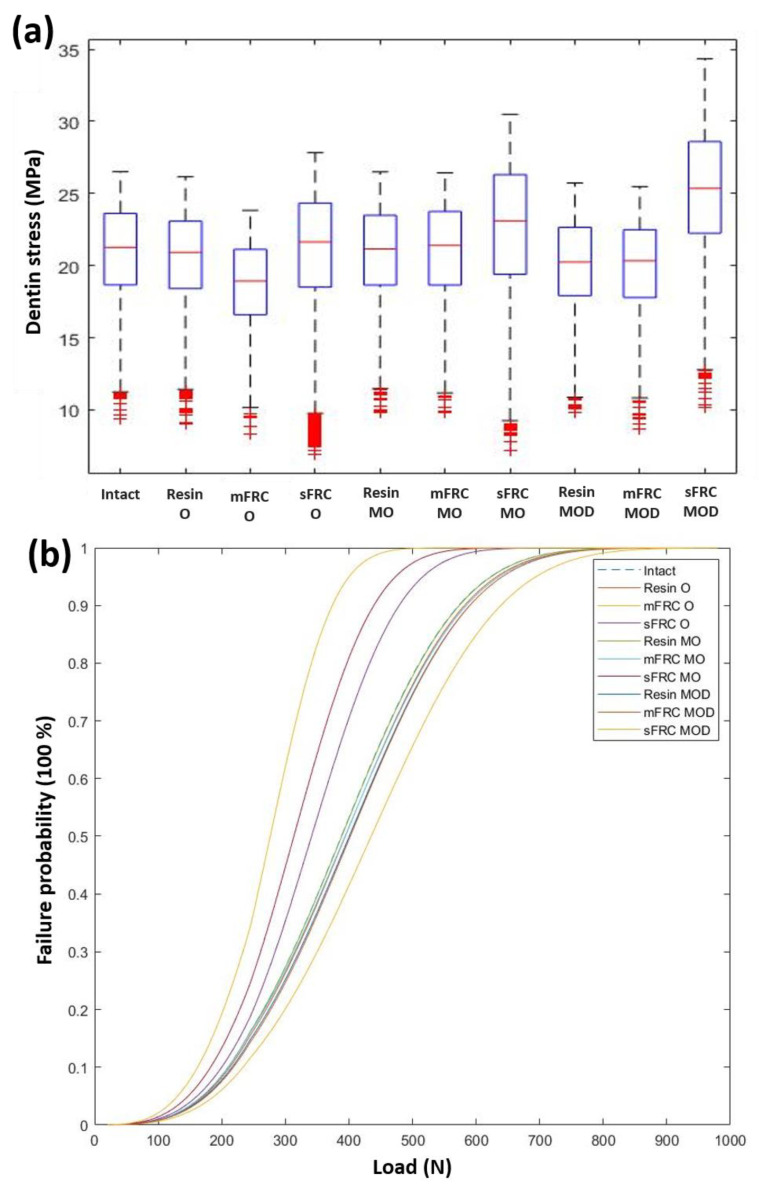
(**a**) Boxplot showing dentin stresses for three types of cavity: occlusal (O), mesio-occlusal (MO) and mesio-occluso-distal (MOD), and different restorative strategies: resin, multiple fiber-reinforced composite (mFRC) and single fiber-reinforced composite (sFRC). (**b**) Failure probabilities for three types of cavity: occlusal (O), mesio-occlusal (MO) and mesio-occluso-distal (MOD), and different restorative strategies: resin, multiple fiber-reinforced composite (mFRC) and single fiber-reinforced composite (sFRC).

G_2O_, G_2MO_ and G_2MOD_ exhibited statistically higher cervical root stresses and resin strains than G_1O_ and G_3O_, G_1MO_ and G_3MO_, G_1MOD_ and G_3MOD_, respectively (*p* < 0.05) with regard to the type of restoration. However, G_3O_ and G_3MO_ displayed significantly higher cervical root stresses and resin strains than G_1O_ and G_1MO_, respectively, while G_3MOD_ displayed lower cervical root stresses and resin strains than G_1MOD_. Dentin failure probability curves for the different restoration strategies showed significantly lower failure chances for G3 than for G2 regardless of the load intensity or the cavity configuration (Figure 7).

## 4. Discussion

The aim of this study was to evaluate if the cavity configuration and the type of post endodontic restoration might have an influence on the fracture resistance, the failure mode and the stress distribution of maxillary premolars by using a combined method of fracture failure test and FEA coupled to WA. The knowledge of the complex relationship between residual walls, type of restoration, fracture resistance, failure mode and stress distribution in ETT may be important in predicting clinical prognosis. The post-endodontic restoration, especially in the posterior areas, aims to improve the mechanical strength of the treated element, prevent unfavorable fractures and restore the anatomy and function of a tooth [37].

In the present study, maxillary premolars were used because they have unfavorable crown/root anatomy. They are subject to a combination of compression (occlusal) and shear (lateral) forces, making them more susceptible to fracture than occlusally loaded molars [43,44]. In contrast to many ex vivo studies on ETTs, this study did not investigate the “worst-case scenario” whereby all residual walls are absent, but it focused on a less frequent situation in which endodontically treated premolars presented an O cavity configuration and on more frequent scenarios in which endodontically treated premolars had a MO and MOD cavity configuration.

Clinically, direct restoration without a post is often used to restore ETT premolars with MO and O cavity configurations. This option is relatively inexpensive and may represent an aesthetic alternative to cuspidal coverage restorations. Whereas for premolars with MOD cavities, the indication is to restore them without a post but with cuspidal coverage [45]. Post placement is reportedly more commonly used for indirect restorations than for direct restorations [46,47].

Clinical failure of a restoration is often due to fatigue; therefore, in the present study all specimens were subjected to thermomechanical aging [48,49]. Other parameters could also affect the results of the ex vivo biomechanical tests, such as the diameter of the spherical stainless-steel antagonist, the direction and the speed of the applied force and whether or not the periodontal ligament is simulated. Indeed, according to different studies [50,51], the direction of the spherical antagonist would affect the distribution and accumulation of stresses on the crown and the roots of a teeth. In fact, an angular direction could lead to increased stresses in the roots, while an axial direction would not lead to any significant stress accumulation. Considering the direction of the chewing forces, an angular application in a fracture resistance test is closer to a real clinical situation. As for the diameter of the sphere, this can lead to stress variations on the crown contact area but not at the root level [51]. In fact, the diameter seems to be a less important parameter. In the present study, the speed of the applied force was 1 mm/min. The literature indicates that a lower speed determines higher plastic deformation and, consequently, higher fracture resistance measurements could be recorded [52].

The simulation of the PDL could lead to an increase in stress concentration, especially in the cervical region of the root, which is one of the most sensitive areas to root fractures [51,53]. For this reason, the PDL presence has been simulated, and a 45° oblique compression force has been used to load the samples.

In previous studies, fracture resistance data have reported a high standard deviation ranging from 17% to 37% [54]. Individual variations in the characteristics of the different teeth, given by age as well as different mesio-distal, bucco-lingual, and apico-coronal dimensions of the tooth may explain these high standard deviations. The standard deviation reported in the different groups of the present study was in the lower range of the usual values, which was probably because of the high level of samples standardization used. According to a recent study, the value of chewing forces varies greatly from one person to another and is higher in men [55]. However, fatigue plays a determinant role in dental fracture, possibly leading to fracture even at low values. The same force value would not cause the fracture of a young element. Physiological forces faced by maxillary premolars are typically between 250 and 290 N. On the other hand, in bruxism patients or when the tooth is subjected to pathological forces, these forces can exceed 770 N [56]. Regardless of the different cavity configurations and materials used, all groups in the present study exceeded these physiological forces [54], while none of the groups exceed the pathological values.

The material used to hold the specimens during the mechanical test should simulate the ability of the bone to absorb the masticatory forces and therefore withstand the load applied. There is no consensus on the ideal material to be used, and it varies from study to study: acrylic resin, as used in the present study, polystyrene resin, resin-reinforced plaster [57,58,59].

According to the results of the current investigation, groups G_1_ and G_2_ restored, respectively, with composite and composite plus conventional fiber post showed statistically lower resistance values compared to the group of intact teeth (G_contr_). On the contrary, G_3_ restored with multifilament fiberglass posts reported no statistical difference compared to the group of intact teeth regardless of the cavity configuration. In addition, this difference in the biomechanical behavior was also confirmed by FEA, which reported a small but significant difference on root stresses between G_contr_ and G_3_ with extremely similar probability in the failure curves.

The null hypothesis that the restorative protocol has no effect on fracture resistance was rejected, since group G_3_ had a statistically higher fracture resistance than groups G_1_ and G_2_ regardless of the cavity configuration. Furthermore, the specimens restored with multifilament fiberglass posts had a fracture resistance and failure probability comparable to that of an intact tooth. These results may be also due because in G_3_, the post-space was not prepared, thus limiting the loss of root dentin during intracanal post preparation [17,30]. This is in accordance with some recent studies showing that the use of multiple fiber posts in oval canals allowed a better stress distribution and strengthened the dental unity [22,29,60]. Furthermore, from a biomechanical point of view, the use of a single post placed in the middle of the post-space along the neutral axis of the tooth would not be optimal [61]. The results of the stress analysis in the present study, instead, suggested that it should be as close as possible to the dentinal walls of the canal, where the tensile stresses are increased [62].

In a multifilament fiberglass posts, the micro-posts may be better distributed in the canal, allowing a better fit with the post-space walls, thus minimizing the negative tensile stresses applied during loading of the restoration [60]. Moreover, when a single round post is used in an oval canal, the mismatch between the diameter of the post and the canal shape represents a clinical challenge [63,64]. In these cases, if the post does not fit well at the coronal level, there will be a thick layer of cement [63,65]. Since the diameter of a micro-post is 0.3 mm, the use of fasciculated micro-posts helps to fill narrow, large or irregular root canals without mechanically preparing them. Clinically, after filling the post-space with resin composite, the fasciculated post is introduced 3 mm lower than the root canal orifice, and then, each individual micro-post is pushed vertically one by one to fill the space. If necessary, additional micro-posts can be added individually to fill remaining gaps.

Regardless of the cavity configuration, no statistical difference in fracture resistance was found between G_1_ and G_2_. This is in disagreement with a previous in vitro study showing that single fiber post restoration had higher fracture resistance than composite restoration without any intra-radicular retention [25]. Nevertheless, these different results might be attributed to the different study design, because in the other study, tests did not simulate the presence of the periodontal ligament, did not perform thermomechanical aging and used an axial compression to load the samples. The null hypothesis that the cavity configuration has no effect on the fracture resistance was partially rejected, since in G_1_, the subgroups G_1O_ and G_1MO_ had a statistically higher fracture resistance than G_1MOD_, while no difference was found between G_1O_ and G_1MO_. As for G_2_, the subgroup G_2O_ had a statistically significantly higher fracture resistance than G_2MOD_, while no difference was shown between G_2O_ and G_2MO_ and between G_2MO_ and G_2MOD_. Concerning the G_3_, no statistical difference was found among G_3O_, G_3MO_ and G_3MOD_. Therefore, these results for groups G_1_ and G_2_ are in partial agreement with previous studies [22,66], which showed that fracture resistance decreased as the volume of residual coronal tooth structure decreased, but they are not in agreement for group G_3_ in which the cavity configuration did not have a statistically significant impact on fracture resistance. Analysis with FEA, a non-destructive method, confirmed that the stress distribution was similar among the different restorative options [67], but that higher stress concentration was reported for the most invasive coronal cavity configuration and post-space preparation. Similar stress distributions were reported in finite element models investigating the use of these micro-fiber posts in incisors [68]. However, the lowest stress values were reported for the use of a standard fiber poIt in addition to micro-fiber posts in comparison to only using micro-fiber posts [68]. This strategy appears to be valuable in cases of incisors where the root canal is often large but should be questioned in cases of maxillary premolars frequently presenting thin root canal walls.

In addition to the numerical value of the fracture resistance, it is important for clinicians to evaluate the mode of failure, above or below the crestal bone, because it can dictate tooth restorability. The G_1_ group showed a favorable fracture pattern in a lower percentage of cases than the G_2_ and G_3_ groups, while G_2_ showed a favorable fracture pattern in a lower percentage of cases than G_3_. Similar results were found in relation to the different cavity configurations. Nevertheless, according to the statistical analyses performed, the third null hypothesis that the restorative protocol had no effect on the fracture pattern was accepted, because no statistical difference among the different groups and subgroups was observed. The fourth null hypothesis that the cavity configuration has no effect on the failure mode may be accepted too, since no statistical difference was showed among the three types of cavity configuration and interaction analysis among subgroups with the same type of post-endodontic restoration. Limitations of the present study are mainly due to the destructive mechanical experimental test used, because its clinical translation must be performed with extreme caution. Moreover, a static compressive force was used; however, the forces in the oral cavity are dynamic, with a constantly changing rate, magnitude and direction. In addition, teeth were collected from patients which have different ages. This difference in age induces changes occurring the dental tissues that could influence the results of our study [69,70]. Further ex vivo studies will be required to investigate whether an indirect restoration may reduce the impact of the different restorative protocols tested in the present study. Furthermore, clinical studies should be conducted to confirm these laboratory findings. Furthermore, a single finite element model was used following the protocols of validated finite element studies [40,71]. It is, however, of particular importance to note that patient-specific parameters such as the root canal anatomy could influence the biomechanical behavior of the tooth [72] and should be investigated in future studies using a higher number of finite element models. Moreover, while several failure criteria were employed to analyze in silico fracture in the damaged premolar, none was identified as the definitive or most reliable approach [40]. This highlights the urgent need for future studies to integrate both in vitro and in silico comparisons to better inform clinical decision making.

## 5. Conclusions

Within the limits of this ex vivo study, multifilament fiberglass posts, without post-space preparation, seem to increase the fracture resistance values up to those of an intact tooth, regardless of the cavity configuration, after thermomechanical aging simulating 5 years of work into the oral cavity. In contrast, no statistically significant difference was found among the groups tested in terms of fracture patterns.

## Figures and Tables

**Table 1:** Mean ± standard deviations of fracture load resistance and the standard deviation for experimental subgroups and G_contr_.

Group	Subgroups			Statistical Analysis (*p* < 0.05)
Cavity Configuration	O	MO	MOD	
No-post (G_1_)	506 ± 74	414 ± 96	307 ± 106	G_1O_ < G_1MOD_ G_1MO_ < G_1MOD_
s-FRC post(G_2_)	507 ± 89	436 ± 135	372 ± 138	G_1O_ < G_1MOD_
m-FGP (G_3_)	724 ± 217	656 ± 118	631 ± 103	No significant difference
Intact premolars (G_Contr_)	688 ± 110			
Statistical analysis (*p* < 0.05)	G_1O_, G_2O_ < G_Contr_ G_1O_, G_2O_ < G_3O_	G_1MO_, G_2MO_ < G_Contr_ G_1MO_, G_2MO_ < G_3MO_	G_1MOD_, G_2MOD_ < G_Contr_ G_1MOD_, G_2MOD_ < G_3MOD_	

## Data Availability

The data presented in this study are available on request from the last author (mancino@unistra.fr).
